# Q Fever During Pregnancy: An Emerging Cause of Prematurity and Abortion

**DOI:** 10.1155/S1064744901000084

**Published:** 2001

**Authors:** Francisco Jover-Díaz, Jaqueline Robert-Gates, Lucio Andreu-Gimenez, Jaime Merino-Sanchez

**Affiliations:** 1 Internal Medicine Department San Juan University Hospital Alicante Spain; 2 C/Amadeo de Saboya 2, 5 B Alicante 03012 Spain

## Abstract

*Background:* Although the pathogenic role of *Coxiella burnetii* infection during pregnancy is controversial, some cases of stillbirth and abortion occurring after an acute or chronic infection have been mentioned in the literature. Recently, Q fever has been advocated as a significant cause of morbidity and mortality in pregnancy

*Case:* We describe an 18-year-old primipara woman admitted to our hospital for high fever and pancytopenia during an acute *C. burnetii* infection. She was successfully treated with clarithromycin, overcoming fever and pancytopenia. Finally, she gave birth to a healthy infant, and 1 year later both remained well.

*Conclusion:* Q fever is a potentially serious disease in pregnancy owing to the possibility of placenta infection and fetal transmission affecting its outcome. Q fever infection should be suspected in unexplained febrile episodes or abortion during pregnancy, when epidemiologic and clinical data are present.We believe that *C. burnetii* serology should be tested in cases of fever of known origin or unexplained abortions, as the TORCH infections are.


### Q fever during pregnancy: an emerging cause of prematurity and abortion

**Francisco Jover-Díaz, Jaqueline Robert-Gates, Lucio Andreu-Gimenez, and Jaime Merino-Sanchez**

*Internal Medicine Department, San Juan University Hospital, Alicante, Spain*

***Background:*** Although the pathogenic role of *Coxiella burnetii* infection during pregnancy is controversial, some cases of stillbirth and abortion occurring after an acute or chronic infection have been mentioned in the literature. Recently, Q fever has been advocated as a significant cause of morbidity and mortality in pregnancy

***Case:*** We describe an 18-year-old primipara woman admitted to our hospital for high fever and pancytopenia during an acute *C. burnetii* infection. She was successfully treated with clarithromycin, overcoming fever and pancytopenia. Finally, she gave birth to a healthy infant, and 1 year later both remained well.

***Conclusion:*** Q fever is a potentially serious disease in pregnancy owing to the possibility of placenta infection and fetal transmission affecting its outcome. Q fever infection should be suspected in unexplained febrile episodes or abortion during pregnancy, when epidemiologic and clinical data are present. We believe that *C. burnetii* serology should be tested in cases of fever of known origin or unexplained abortions, as the TORCH infections are.

Key words: *COXIELLA BURNETII*; STILLBIRTH; INTRAUTERINE DEATH

Q fever is a worldwide zoonosis caused by a Gram-negative obligate intracellular bacterium, *Coxiella burnetii*. Frequently, it is a self-limited febrile episode or occurs in association with pneumonia[1]. Both domesticated and wild animals have been demonstrated to have reservoirs of the infection in their placenta, which acts as the source of contamination via the aerosol route at the time of parturition[2]. Here we describe a woman pregnant for 20 weeks admitted to our hospital with fever and pancytopenia during an acute *C. burnetii* infection.

An 18-year-old primipara woman was hospitalized because of high fever and chills during the previous week. She lived in the countryside and worked as a farmer. Physical examination revealed a pale patient with fever (temperature 39°C). No other remarkable data were detected. Laboratory studies showed a marked pancytopenia (hemoglobin 8.62 g/dl, white blood cell count (WBC) 3250 cell/ml, and platelet count 90 400 cell/ml). Other laboratory results are shown in Table 1. Microbiologic cultures from blood, urine and amniotic fluid were aseptic. There was no serologic evidence for recent infection with *Salmonella,* Epstein–Barr virus, cytomegalovirus, *Brucella,* human immunodeficiency virus (HIV), syphilis, toxoplasma, B-19 parvovirus, *Borrelia burgdorferi, Coxiella burnetii, Rickettsia conorii* or *Leishmania.* Chest radiograph and abdominal echography yielded normal findings. She received empirical antibiotic therapy with ampicillin, gentamicin and clindamycin, but fever continued.

Correspondence to: Francisco Jover Díaz, C/Amadeo de Saboya 2, 5 B, 03012 Alicante, Spain. E-mail: fjoverdiaz@coma.es

**Table 1** Results of laboratory tests

| *Test* | *Value* |
|---|---|
| Erythrocyte sedimentation rate | 50 mm/h |
| C-reactive protein level | 5.8 mg/dl |
| Hemoglobin level | 8.62 g/dl |
| Reticulocyte counts | 1% |
| Platelet counts | 90 400/ml |
| White blood cell (WBC) count | 3250 ml |
| Alanine aminotransferase level | 370 U/l |
| Aspartate aminotransferase level | 467 U/l |
| γ-Glutamyltransferase level | 23 U/l |
| Alkaline phosphatase level | 240 U/l |
| Bilirubin level | 0.7 mg/dl |
| Lactate dehydrogenase level | 725 U/l |
| Antinuclear antibodies | Negative |
| Anti-DNA antibodies | Negative |

Pancytopenia and liver damage progressively increased, so a twice-per-day clarithromycin regimen (500 mg) was initiated. Forty-eight hours later, fever disappeared and hematologic and biochemical parameters became normal. One month later, a significant rise in the indirect immunofluorescence (IFI) phase II antibody titers (1/512) of *C. burnetii* was detected, and retrospectively an acute Q fever was diagnosed. After 40 weeks of pregnancy, she gave birth to a healthy child. Now, 1 year later, the patient and her baby are well.

Maternal infection may present as acute Q fever or chronic uteroplacental infection reactivated by the immunocompromised state associated with pregnancy. Although the pathogenesis of fetal disease is less well known, immunocomplex formed may cause vasculitis and vascular thrombosis occasioning placental insufficiency, responsible for prematurity and abortion. Fetal direct infection has been proved in some cases in which *C. burnetii* was isolated from several fetal viscera[2].

Recently, Stein and Raoult collected all cases published until 1998, demonstrating that Q fever can be a significant cause of morbidity and mortality in pregnancy[2]. In only five of the 23 cases reported was a healthy child born. There were eight cases of premature birth, eight cases of abortion and five cases of neonatal death within 2 days. Diagnosis was made by serology in all cases, demonstrating an acute infection in 14 and a typical chronic profile in nine. In 12 of 17 cases, *C. burnetii* has been identified in the placenta by culture. There was a case of nosocomial transmission of the disease to the obstetrician who took care of the birth, caused by aerosolization of the microorganism from the infected placenta[3].

With respect to treatment, although many controlled clinical trials comparing antibiotics have been performed, clinical experience seems to accord with *in vitro* susceptibility[4]. It seems that in cases of acute infection a bacteriostatic effect is sufficient, whereas in cases of chronic infection a bactericide effect is necessary. The selection of an antimicrobial treatment in pregnancy is controversial. Tetracycline, the first-choice agent, has been related to maternal hepatotoxicity, fetal tooth discoloration and retarded skeletal bone maturity. Macrolides have a bacteriostatic effect comparable to that of other antibiotics and might be an alternative[5]. Although the efficacy of quinolones against *C. burnetii* has not yet been established, ciprofloxacin has shown *in vitro* susceptibility testing comparable or superior to that of conventional drugs. In addition, it crosses the placenta and is safe in pregnancy[4]. Nevertheless, because the activity of antibiotics is modest, it seems reasonable, mainly in cases of chronic infection, to associate several drugs by analogy with endocarditis, in which the synergic association of doxycycline and rifampicin or ciprofloxacin has reduced mortality[6].

Hematologic complications of Q fever occur infrequently and include hemolytic anemia from cold agglutinins and thrombocytopenia associated with endocarditis. We emphasize the severe pancytopenia in our case that yielded to clinical improvement. This complication has been attributed to a hemophagocytic syndrome. Hemophagocytosis is an unusual cause of increased consumption of mature sanguineous cells secondary to neoplasic or infectious conditions. Two cases of hemophagocytosis in nonpregnant patients have been described as a complication of acute Q fever[7,8]. Bacterial lipopolysaccharides and some cytoquines have been related to activation of macrophages and secondary phagocytosis. Only one of the 23 cases diagnosed during pregnancy presented with pancytopenia suggestive of hemophagocytosis.

In summary, Q fever is a potentially serious disease in pregnancy because of the possibility of placenta infection and fetal transmission. It is advisable for pregnant women to avoid parturient animals, including domestic ones. Diagnosis is helpful not only for the high risk of prematurity and abortion but also for the clinicians attending delivery (nosocomial transmission). Because maternal incidence might be higher than expected[2], Q fever should be suspected in cases of fever of unknown origin during pregnancy or unexplained abortion when epidemiologic and clinical data are concordant. We think that *C. burnetii* serology should be tested and repeated for seroconversion in unexplained fever or abortions, as the TORCH infections are.

### REFERENCES

1. Tellez A, Sanz J, Valkova D, *et al.* Q fever in pregnancy: case report after a 2-year follow-up. *J Infect* 1998;37:79–81
2. Stein A, Raoult D. Q fever during pregnancy: a public, health problem in southern France. *Clin Infect Dis* 1998;27:592–6
3. Raoult D, Stein A. Q fever during pregnancy – a risk for women, fetuses, and obstetricians. *N Engl J Med* 1994;330:371
4. Ludlam H, Wreghitt TG, Thornton S, *et al.* Q fever in pregnancy. *J Infect* 1997;34:75–8
5. Gikas A, Spyrudaky I, Psaroulaki A, *et al.* In vitro susceptibility of *Coxiella burnetii* to trovafloxacin in comparison with susceptibilities to pefloxacin, ciprofloxacin, ofloxacin, doxycycline, and clarithromycin. *Antimicrob Agents Chemother* 1998;42: 2747–8
6. Levy PY, Drancourt M, Etiene J, *et al.* Comparison of different antibiotic regimens for therapy of Q fever endocarditis. *Antimicrob Agents Chemother* 1991;35:533–7
7. Hufnagel M, Niemayer C, Zimmerhackl LB, *et al.* Hemophagocytosis: a complication of acute Q fever in a child. *Clin Infect Dis* 1995;21:1029–31
8. Estrov Z, Bruck R, Shtalrid M, *et al.* Histiocytic hematophagocytosis in Q fever. *Arch Pathol Lab Med* 1984;108:7

RECEIVED 8/14/00; ACCEPTED 11/30/00
